# Clinical spectrum and outcomes of anti-metabotropic glutamate receptor 5 encephalitis in Chinese patients: a case report and literature review

**DOI:** 10.3389/fimmu.2025.1656832

**Published:** 2025-11-06

**Authors:** Zhenyu Niu, Siwei Chen, Jianchun Wang, Meng Yu, Jingru Ren, Ran Liu, Jing Guo, Nan Zhang, Feng Gao, Hongjun Hao

**Affiliations:** 1 Department of Neurology, Peking University First Hospital, Beijing, China; 2 Rare Disease Medical Center, Peking University First Hospital, Beijing, China

**Keywords:** anti-mGluR5 encephalitis, metabotropic glutamate receptor 5, autoimmune encephalitis, neuroimmune disease, glucococorticoids

## Abstract

**Background:**

Anti-metabotropic glutamate receptor 5 (mGluR5) encephalitis is a rare autoimmune disorder that targets the metabotropic glutamate receptor. It is frequently linked to limbic encephalitis and paraneoplastic syndromes, such as Ophelia syndrome associated with Hodgkin lymphoma. Due to its rarity, the complete clinical spectrum and regional variations of this condition, particularly among Chinese populations, remain inadequately understood.

**Case description:**

We present a case of a 39-year-old Chinese male diagnosed with anti-mGluR5 encephalitis. The patient initially presented with persistent fever, which later progressed to seizures, psychosis, apathy, drowsiness, and memory impairment. Brain imaging findings were unremarkable, while electroencephalogram (EEG) revealed predominant beta wave activity. Cerebrospinal fluid (CSF) analysis showed pleocytosis and elevated protein levels. Both serum and CSF tested strongly positive for mGluR5 antibodies (titers 1:160 and 1:640, respectively), with no other autoantibodies detected. A thorough evaluation revealed no evidence of an underlying tumor. Symptom resolution was rapid following intravenous methylprednisolone pulses, with sustained remission achieved through rituximab therapy combined with a gradual tapering of steroids over one year of follow-up.

**Literature review:**

In 18 Chinese patients, the median age was 36 years (range 7–60), with 61% being male (11/18). Only 17% (3/18) had associated tumors, including two cases of teratomas and one of gangliocytoma. The clinical manifestations were highly diverse, with headache (44.4%), irritability/sleepiness (38.9%), and seizures (38.9%) being the most prevalent symptoms. Additional symptoms included hallucinations (33.3%), fever (27.8%), memory impairment (27.8%), dystonia (22.2%), and consciousness disorders (22.2%). Antibody analysis showed that 94.4% (17/18) of patients were serum mGluR5-positive, 61.1% (11/18) had mGluR5 antibodies in cerebrospinal fluid, and 55.5% (10/18) tested positive in both. Magnetic resonance imaging (MRI) anomalies were identified in 72.2% of patients, typically affecting the temporal/insular lobes and deep gray matter. Immunotherapy, consisting of steroids, intravenous immunoglobulin and/or immunosuppressive drug, was administered to 94.4% (17/18) of patients, resulting in favorable outcomes for 17 cases.

**Conclusion:**

This study highlights that anti-mGluR5 encephalitis in Chinese patients exhibits significant clinical heterogeneity and a notably low association with tumors (17%), contrasting with higher rates reported elsewhere. Both serum and CSF antibody testing are crucial for diagnosis. Immunotherapy, including steroids and potentially rituximab, appears highly effective. Clinicians should be aware of the broad symptom spectrum and the relatively low paraneoplastic risk in this population. Vigilance for tumors, especially teratomas and neural tumors, remains essential.

## Introduction

Anti-mGluR5 encephalitis is a rare autoimmune neurological disorder characterized by antibodies targeting the metabotropic glutamate receptor 5 (mGluR5), a crucial receptor involved in synaptic plasticity and memory formation (1, 2). Clinically, this condition presents with a wide range of neuropsychiatric, cognitive, and motor symptoms. Common manifestations include memory deficits, seizures, behavioral disturbances, psychosis, and movement abnormalities, though the presentation can vary significantly among patients (3–5). The disease has been linked to diverse clinical phenotypes and triggers, including paraneoplastic syndromes like Ophelia syndrome in Hodgkin lymphoma, as well as idiopathic and post-infectious causes (1, 2, 6). Here we report a case of a male presenting with limbic encephalitis characterized by seizures and psychosis, with confirmed mGluR5 antibody positivity. Additionally, we summarize the clinical characteristics, treatment strategies, and prognosis of previously reported Chinese cases of anti-mGluR5 encephalitis. This analysis aims to identify common disease patterns, explore potential regional disparities, and deepen understanding to advance future diagnosis and treatment approaches.

## Case description

A 39-year-old man with no significant medical or family history presented with intermittent episodes of unconsciousness and limb convulsions following a month-long persistent fever. During these episodes, he exhibited sudden loss of consciousness, rightward head deviation, facial and lip cyanosis, clenched jaw, and generalized tonic-clonic movements, accompanied by an absence of gaze fixation. Each episode lasted approximately three minutes, after which he regained consciousness but had no recollection of the events. As the episodes became more frequent, he gradually developed apathy, drowsiness, memory impairment, disorientation, and reduced appetite. One week later, due to increasing agitation and an incident of physical aggression, he was admitted to a psychiatric hospital, where his symptoms were temporarily managed with haloperidol and olanzapine. He was subsequently referred to our hospital for a thorough evaluation of potential organic causes. The patient, a mechanic by profession, had a 20-year history of heavy smoking, frequently worked night shifts, and consumed alcohol occasionally, though he reported no history of excessive drinking prior to symptom onset.

**Figure 1:**
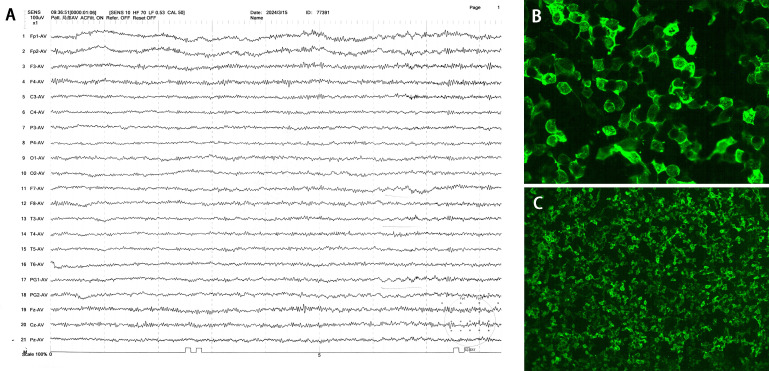
EEG and CSF mGluR5 antibodies of the patient. **(A)** EEG performed on day 1 after admission showed predominantly β-wave patterns with short β rhythms. Time and amplitude scale bars are indicated. **(B, C)** The detection of CSF mGluR5 antibodies was carried out by a cell-based assay that involved human embryonic kidney (HEK) 293 cells that over-expressed the mGluR5 (×200, ×40).

Upon admission, the patient was conscious and able to recognize relatives but exhibited incoherent speech, emotional agitation, and restlessness, which hindered cooperation during the physical examination. Muscle strength was assessed as normal. Midazolam infusion was initiated to maintain continuous sedation. Initial laboratory tests revealed an elevated white blood cell count of 13.98×10^9/L, predominantly neutrophils (84.8%), alongside increased C-reactive protein levels at 73.28 mg/L. Liver and kidney function tests, as well as urinalysis, were largely unremarkable. However, creatine kinase levels were elevated to 836 IU/L, while blood ammonia and thyroid function remained within normal limits. A 20-minute short-term electroencephalogram (EEG) predominantly displayed β-wave patterns, interspersed with brief β rhythm bursts and occasional θ waves, most prominent in the left anterior-mid temporal region and sphenoidal electrodes (**Figure 1A**). During the EEG, the patient was alert without any seizures. Head CT scans revealed no significant abnormalities. Agitation prevented the patient from cooperating with a contrast-enhanced MRI scan; consequently, only T1-weighted, T2-weighted, and diffusion-weighted sequences were obtained, none of which showed significant abnormalities. To rule out concomitant peripheral neuropathy, nerve conduction velocity and electromyogram examinations were performed and revealed no significant abnormalities.

In conclusion, the patient was initially diagnosed with encephalitis. To determine the underlying etiology, a lumbar puncture was performed on the second day of admission. The opening pressure was 150 mmH_2_O. CSF analysis revealed a nucleated cell count of 67/mm³, predominantly mononuclear cells (98.5%), along with an elevated protein concentration of 0.41 g/L. Glucose and chloride levels were within normal limits. Gram staining, as well as bacterial and fungal cultures, in addition to multiple next-generation sequencing (NGS) tests, yielded negative results. Serum and CSF neuroimmunological examinations identified positive oligoclonal bands (OCB) in CSF, classified as Pattern 2. The CSF albumin quotient was elevated to 13.02 × 10^-3, with an immunoglobulin G (IgG) Index of 0.88 and a 24-hour intrathecal IgG synthesis rate of 9.45 mg/24h. Using the cell-based assay (CBA) method, CSF mGluR5 antibodies demonstrated strong positivity with a titer of 1:640 (**Figures 1B, C**), while serum mGluR5 antibodies were also positive at a titer of 1:160. Conversely, tests for serum and CSF antibodies related to aquaporin 4 (AQP4), myelin oligodendrocyte glycoprotein (MOG), glial fibrillary acidic protein (GFAP) were negative. While all other tested antibodies related to encephalitis and paraneoplastic syndrome, including those against N-methyl-D-aspartic acid receptor (NMDAR), α-amino-3-hydroxy-5-methyl-4-isoxazole-propionic acid receptor (AMPAR), and Leucine-rich glioma inactivated 1 (LGI1) were all negative (for the complete list, see **Supplementary Table S1**).

**Figure 2:**
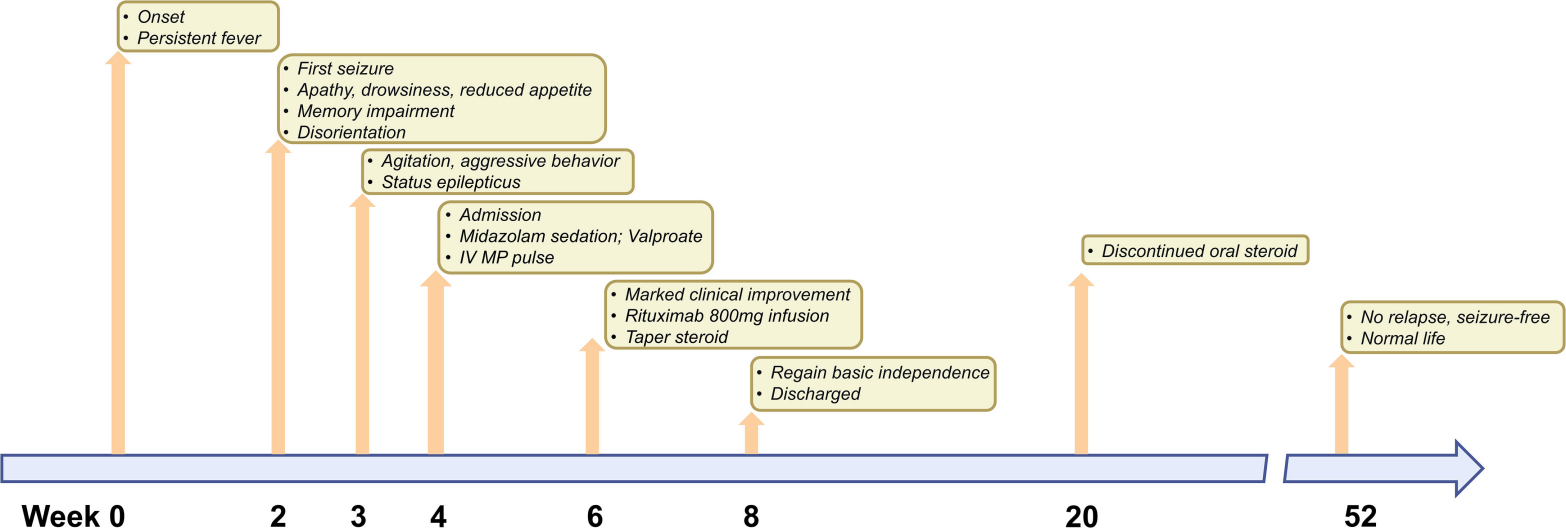
Timeline of complications or clinical manifestations after onset and corresponding treatment options. The time of the patient’s onset is marked as week 0. The time of the patient’s last follow-up is marked as week 56. At the top of the timeline, treatment options for complications or symptoms that occurred at different times are indicated.

After admission, the seizure frequency continued to increase, evolving into recurrent generalized tonic-clonic seizures without full recovery of consciousness in between, meeting the diagnostic criteria for status epilepticus. To manage this critical condition, an intravenous loading dose of valproate (30 mg/kg) was administered, followed by a maintenance dose of 1500 mg daily. Concurrently, the infusion rate of midazolam was increased. Being diagnosed with mGluR5-encephalitis, the patient was treated with high-dose intravenous methylprednisolone (MP) pulses, receiving 1000 mg/day for the initial three days, followed by 500 mg/day for the next three days. Subsequently, oral prednisone was started at a dosage of 2 mg/kg/day. His seizures were gradually brought under control with this regimen, with marked clinical improvement was observed after two weeks of treatment. A follow-up lumbar puncture revealed the absence of nucleated cells in the cerebrospinal fluid (CSF), with protein levels measured at 0.43 g/L. The patient then received an 800 mg infusion of rituximab as part of B-cell depletion therapy and was discharged from the hospital. One year post-discharge, the patient maintained a stable condition, leading a normal life without recurrence of symptoms or seizures. Oral prednisone had been discontinued for four months, and no second dose of rituximab had been administered. Timeline of the patient’s clinical manifestations and corresponding treatment options was summarized in **Figure 2**.

## Literature review

We collected and reviewed all case reports on anti-mGluR5 encephalitis published in English before May 2025 through searches on PubMed, Web of Science, and ScienceDirect. A total of 18 cases involving Chinese individuals were identified, comprising eight independent case reports and 10 cases detailed in two case series (1, 3, 6–13). For further analysis, we gathered essential information, including clinical features, results from auxiliary examinations, tumor associations, treatment responses, and clinical outcomes (**Table 1**). Examination results encompassed CSF analysis, MRI findings, EEG results, and tests for other autoantibodies (**Table 2**).

**Table 1:** Basic information, symptoms, tumor associations, treatment and clinical outcomes of 18 patients.

Pt. no.	Age	Gender	Symptoms on admission	Tumor complicated	Intervention	Response time to recovery (weeks)	Status at last follow up total follow up after discharge(weeks)	
1	12	Male	• Headache, fever, N/V • Memory impairment • Irritability, sleepiness • Hallucinations	Retroperitoneal gangliocytoma	• Tumor resection • IV Ig 0.4g/kg/d x 5d • IV MP pulse • Steroid taper	• Resolved • 4 weeks	• Clinical Response: Relapsed after 1 month due to poor medication compliance, resolved after IVIg • Total follow up ~70weeks
2	7	Female	• Seizures • Dystonia • Self-talk	Shwachman-Diamond syndrome(SDS), after HSCT	• IV Ig 0.4g/kg/d x 5d • IV MP pulse • Steroid taper • Levetiracetam • Trihexyphenidyl	• Resolved • NM	• Clinical Response: Remained symptom-free • SBDS gene mutation negative • Total follow up ~72weeks
3	44	Male	• Headache, N/V • Blurred vision • Dysphagia, dysarthria • Limb pains and weakness • Urinary retention	No tumor	• IV Ig 0.4g/kg/d x 5d	• Resolved • 4 weeks	• Clinical Response: Completed resolved with no tumor found • Total follow up ~24weeks
4	38	Male	• Headache, N/V • Aphasia • Irritability • Status Epilepticus	No tumor	• IV Ig 0.4g/kg/d x 5d • IV MP pulse • Steroid taper • Oral MMF 1g/d	• Resolved • 4 weeks	• Clinical Response: Remained symptom-free • Total follow up ~24weeks
5	19	Female	• Anxiety, sweating • Dystonia • Weight loss	Left ovarian teratoma, 8 years after oophorectomy without relapse	• IV Ig 0.4g/kg/d x 5d • IV MP pulse • Steroid taper • Escitalopram	• Resolved • 6 weeks	• Clinical Response: Remained symptom-free • Total follow up ~78weeks
6	60	Female	• Headache • Blurred vision	No tumor	• IV MP pulse • Steroid taper • Oral MMF 1g/d	• Resolved • 2 weeks	• Clinical Response: Sight restored • Total follow up ~8weeks
7	65	Female	• Doc • Status Epilepticus • Dystonia	No tumor	• IV Ig 0.4g/kg/d x 5d • IV MP pulse • Steroid taper • Levetiracetam, valproate	• Resolved • 2 weeks	• Clinical Response: Remained symptom-free • Total follow up ~14weeks
8	29	Male	• Headache, fever, Doc • Fatigue, anorexic • Vertigo, ataxia • Irritability, hiccough	No tumor	• IV Ig 0.4g/kg/d x 5d • IV MP pulse • Steroid taper	• Resolved • 3 weeks	• Clinical Response: Remained symptom-free and antibodies were negative • Total follow up ~26weeks
9	52	Male	• Fever, N/V, diarrhea • Cognitive decline • Status Epilepticus • Aphasia	No tumor	• IV MP 40mg/d x 14d	• Resolved • 2 weeks	• Clinical Response: Mildly recovered • Total follow up ~4weeks
10	22	Female	• Memory impairment • Sleepiness	No tumor	• IV MP 80mg/d x 21d • Steroid taper	• Resolved • 4 weeks	• Clinical Response: Remained symptom-free • Total follow up ~21weeks
11	36	Male	• Aphasia • Status Epilepticus	No tumor	• IV Ig 0.4g/kg/d x 5d • Steroid	• Resolved • NM	• Clinical Response: Significantly recovered without seizures • Total follow up ~26weeks
12	51	Male	• Personality changes • Cognitive decline • Hallucinations, delusions, sleepiness	No tumor	• IV MP pulse • Steroid taper	• Resolved • NM	• Clinical Response: Remained stable mental state • Total follow up ~18weeks
13	58	Male	• Memory impairment • Absence seizures	No tumor	• Oral steroid taper	• Resolved • 8 weeks	• Clinical Response: Frequency of seizures reduced • Total follow up ~8weeks	
14	32	Male	• Headache, fever • Personality changes • Memory impairment • Irritability, sleepiness • Hallucinations	No tumor	• IV Ig 0.4g/kg/d x 5d • IV MP pulse • Steroid taper • Oral MMF	• Resolved • NM	• Clinical Response: Partial recovery, stable after 2 relapses • Total follow up ~180weeks	
15	36	Male	• Headache, • Blurred vision • Personality changes • Irritability, sleepiness • Hallucinations	No tumor	• IV Ig 0.4g/kg/d x 5d • IV MP pulse • Steroid taper	• Resolved • NM	• Clinical Response: Remained symptom-free • Total follow up ~104weeks	
16	35	Female	• Headache, Doc • Prosopagnosia • Memory impairment • Seizures, dystonia	Mature teratoma	• Tumor resection • IV Ig 0.4g/kg/d x 5d • IV MP pulse • Steroid taper	• Partial resolved • NM	• Clinical Response: Dead after 6 months • Total follow up ~26weeks	
17	35	Female	• Fever, depression • Executive dysfunction • Hallucinations	No tumor	• IV MP pulse • Steroid taper	• Resolved • NM	• Clinical Response: Remained symptom-free • Total follow up ~78weeks	
18	59	Male	• Diarrhea, Doc • Aphasia • Hallucinations • Irritability, sleepiness	No tumor	• IV MP pulse	• Resolved • NM	• Clinical Response: Partial recovery • Total follow up ~48weeks	

Doc, Disorders of consciousness; d, days; HSCT, Hematopoietic stem cell transplantation; Ig, Immunoglobulins; IV, Intravenous; MMF, Mycophenolate mofetil; MP, methylprednisolone; NM, Not mentioned; N/V, Nausea and vomiting; SBDS gene, The Shwachman-Bodian Diamond syndrome-associated gene.

**Table 2:** Examination results of 18 patients.

Pt. No.	Brain MRI	Lumbar puncture			Anti mGluR5 antibody	Other auto-antibodies	EEG	Others
		Cell count (cells/µL)	Protein (g/L)	OCB				
1	Right insular lobe T2/FLAIR hyperintensities with contrast enhancement	90	WNL	Pattern 4	Serum: Positive 1:100 (CBA) CSF: Positive 1:3.2 (CBA)	Negative	WNL	High CSF and serum IgG
2	WNL	1	0.18	Negative	Serum: Positive 1:1000 (IIF) CSF: Negative (IIF)	Negative	Increased slow waves during wakefulness, paroxysmal multi-spike slow waves during sleep	Low CD4^+^T cells and natural killer cells
3	WNL	0	1.62	Pattern 4	Serum: Positive 1:30 (CBA) CSF: Positive 1:10 (CBA)	Negative	WNL	NCV showed symmetrical motor and sensory fibers demyelination
4	Left cerebral cortex T2/FLAIR/DWI hyperintensities	396	1.417	NM	Serum: Positive 1:10 (CBA) CSF: Positive 1:10 (CBA)	Positive CSF and serum anti-MOG, CSF anti-NMDAR	Left frontal and temporal lobes diffuse sharp-slow wave activity	
5	WNL	WNL	NM	Pattern 2	Serum: Positive 1:1000 (IIF) CSF: Positive 1:32 (IIF)	Negative	WNL	High CSF IgG
6	Left lateral ventricle and left optic nerve T2/FLAIR/DWI hyperintensities	1	0.23	NM	Serum: Positive 1:32 (CBA) CSF: Negative (CBA)	Positive serum anti-MOG	NM	VEP showed prolonged latency of P100 bilaterally
7	Right putamen and caudate nucleus T2/FLAIR/DWI hyperintensities	WNL	WNL	NM	Serum: Positive 1:10 (IIF) CSF: Negative (IIF)	Positive CSF and serum anti-LGI1	Following myogenic artifact associated with faciobrachial dystonic seizure	CTP showed hyperperfusion in the right basal ganglia
8	Splenium of the corpus callosum T2/FLAIR /DWI hyperintensities	222	3.305	NM	Serum: Positive 1:10 (CBA) CSF: Positive 1:10 (CBA)	Negative	Increased slow waves	Onset 3 weeks after received SARS-CoV-2 vaccination
9	Left temporal, occipital and insula lobes T2/FLAIR hyperintensities	19	NM	NM	Serum: Positive 1:10 (CBA) CSF: Positive 1:100 (CBA)	Negative	Continuous medium-amplitude spikes in the left posterior temporal and occipital regions	Onset 2 weeks after HSV encephalitis treated by IVIg, DXM and acyclovir
10	Bilateral basal ganglia, insula and medial temporal lobes T2/FLAIR hyperintensities	7	NM	NM	Serum: Positive 1:32 (CBA) CSF: Positive 1:10 (CBA)	Negative	NM	
11	WNL	2	NM	Pattern 2	Serum: Positive 1:10 (CBA) CSF: Negative (CBA)	Negative	Spikes in bilateral frontal regions	
12	Bilateral medial temporal lobes T2/FLAIR hyperintensities	16	NM	NM	Serum: Positive 1:10 (CBA) CSF: Negative (CBA)	Negative	NM	
13	Bilateral hippocampus and left insula lobe T2/FLAIR hyperintensities	1	NM	NM	Serum: Positive 1:100 (CBA) CSF: Negative (CBA)	Negative	Spikes and slow waves primarily in the left anterior temporal and sphenoidal regions	
14	Bilateral hippocampus T2/FLAIR hyperintensities	<5	NM	NM	Serum: Positive 1:320 (CBA) CSF: Positive 1:10 (CBA)	Negative	WNL	MRI at onset and at first relapse were normal
15	Right medial temporal lobe, cerebral peduncle, thalamus, and putamen T2/FLAIR hyperintensities	80	NM	Negative	Serum: Negative (CBA) CSF: Positive 1:10 (CBA)	Positive serum anti-Recoverin	WNL	
16	Bilateral hippocampus T2/FLAIR hyperintensities	120	NM	NM	Serum: Positive 1:10 (CBA) CSF: Positive 1:100 (CBA)	Positive CSF and serum anti-NMDAR, CSF anti-AMPAR1&2	Focal slow waves increased and epileptiform discharge	
17	WNL	<5	NM	NM	Serum: Positive 1:10 (CBA) CSF: Negative (CBA)	Negative	WNL	
18	Diffuse dura mater T1WI contrast enhancement	<5	NM	Negative	Serum: Positive 1:100 (CBA) CSF: Positive 1:10 (CBA)	Negative	Increased slow waves	

AMPAR, α-amino-3-hydroxy-5-methylisoxazole-4- propionic acid receptor; CBA, Cell based assay; CSF, Cerebral spinal fluid; CTP, Computed tomography perfusion; DWI, Diffusion weighted imaging; DXM, Dexamethasone; EEG, Electroencephalogram; FLAIR, Fluid attenuated inversion recovery; IIF, Indirect immunofluorescence; IVIg, Intravenous immunoglobulin; LGI1, Leucine-rich glioma inactivated 1; NCV, Nerve conduction velocity; NM, Not mentioned; NMDAR, N-methyl-D aspartate receptor; OCB, Oligoclonal bands; VEP, Visual evoked potential; WNL, Within normal limits.

Among the 18 patients, 11 were male and 7 were female, with ages ranging from 7 to 60 years and a median age of 36 years. Following analysis, it was unexpectedly found that among 18 patients, only 3 had tumors. Specifically, 2 female patients had a history of teratoma, while a male patient was diagnosed with gangliocytoma. Additionally, one patient suffered from Shwachman-Diamond syndrome (SDS) and developed encephalitis following hematopoietic stem cell transplantation (HSCT). The remaining 14 patients had no history of tumors, nor were any suspicious tumors identified during treatment or throughout the follow-up period, which ranged from 4 to 180 weeks.

**Figure 3:**
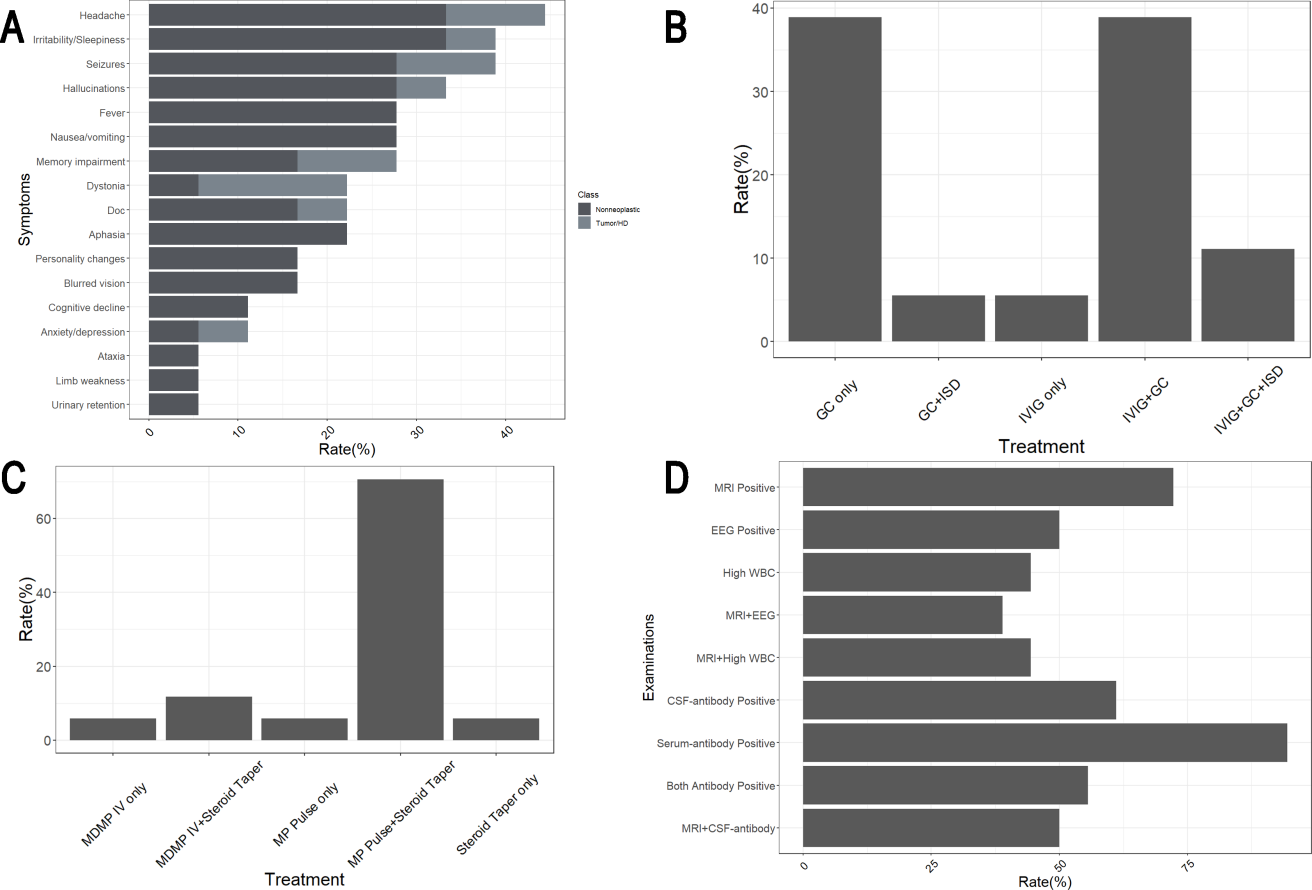
Clinical features of 18 Chinese anti-mGluR5 encephalitis patients reported. **(A)** Symptoms of patients and occurrence rates. Doc, Disorders of consciousness. **(B)** Treatment of 18 patients. GC, Glucocorticoid; IVIg, Intravenous Immunoglobulins; ISD, Immunosuppressive drug. **(C)** Various therapeutic approaches were used among the 17 patients underwent steroid treatment. MDMP, Medium dose methylprednisolone (40mg/d for 1–2 weeks); IV, Intravenous; MP, Methylprednisolone. **(D)** mGluR5 antibodies and other examinations of patients. EEG, Electroencephalogram; WBC, White blood cell; CSF, Cerebral spinal fluid.

An analysis of patients’ admission symptoms highlighted marked heterogeneity among the 18 individuals, with no single symptom occurring in more than 50% of the patients (**Figure 3A**). The most common symptom was headache, observed in 44.4% (8/18) of cases, followed by irritability/sleepiness and seizures, each with an incidence of 38.9% (7/18). Among other typical symptoms of encephalitis, fever and nausea/vomiting—indicative of elevated intracranial pressure—were less frequent, noted in 27.8% (5/18) of patients. Hallucinations, primarily auditory, were present in 33.3% (6/18) of cases. Disorders of consciousness were identified in only 22.2% (4/18) and were associated with more severe illness. Symptoms such as memory impairment, personality changes, and cognitive decline, representing higher cortical dysfunction, occurred at rates of 27.8% (5/18), 16.7% (3/18), and 11.1% (2/18). A noteworthy finding was that dystonia manifested in 4 patients, a common feature of various movement disorders. Among these, 3 patients also had tumor-related or hematological diseases. Rare symptoms were observed as well: one patient experienced typical vestibular system impairments, such as vertigo and ataxia. Another patient displayed acute-onset peripheral nervous system involvement resembling Guillain-Barré syndrome, characterized by limb pain, limb weakness, and urinary retention, with electromyography results indicated symmetrical demyelination of motor and sensory fibers.

In terms of diagnosis, as previously mentioned, Patient 3 was diagnosed with acute inflammatory demyelinating polyradiculoneuropathy (AIDP) due to the presence of typical acute symmetrical peripheral neuropathy symptoms. Additionally, four other patients were found to have positive CNS autoantibodies. Among them, Patient 7 tested positive for CSF and serum anti-LGI1 antibodies, while Patient 16 showed positive results for CSF and serum anti-NMDAR and anti-AMPAR1&2 antibodies. Both were diagnosed with antibody-mediated autoimmune encephalitis involving multiple antibodies. Patient 6 exhibited positive serum anti-MOG antibodies, and Patient 4 tested positive for CSF and serum anti-MOG as well as CSF anti-NMDAR antibodies. Based on clinical and imaging findings, these two patients were diagnosed with autoimmune encephalitis accompanied by CNS demyelinating disease. Among the 18 patients studied, 61.1% (11/18) were CSF mGluR5 antibody positive, 94.4% (17/18) were serum mGluR5 antibody positive, and 55.5% (10/18) were positive for mGluR5 antibodies in both serum and CSF (**Figure 3D**). Of the patients with distinct phenotypes or other positive CNS autoantibodies mentioned earlier, Patient 3, Patient 4, and Patient 16 were positive for mGluR5 antibodies in both serum and CSF, whereas Patient 6 and Patient 7 were positive for serum mGluR5 antibodies only.

Examinations, apart from antibody testing, revealed that 72.2% (13/18) of patients exhibited MRI abnormalities, primarily characterized by patchy T2/FLAIR/DWI hyperintensities with or without T1WI contrast enhancement. These abnormalities were most commonly observed in the temporal and insular lobes, especially in the hippocampus, but could also involve deep structures such as the putamen, caudate nucleus, thalamus, and corpus callosum. EEG abnormalities were detected in 50.0% (9/18) of patients, presenting as increased slow waves and various epileptiform discharges. Elevated CSF WBC counts were found in 44.4% (8/18) of patients, with some cases showing raised CSF protein levels or positive OCB (Pattern 2/Pattern 4). However, due to the limited mention of these findings in most case reports, precise statistics remain unavailable. Notably, all patients with elevated CSF WBC counts and the majority (8/9) of those with EEG abnormalities also demonstrated MRI abnormalities (**Figure 3D**).

2 patients with newly diagnosed tumors underwent tumor resection. Patient 3, who exhibited peripheral neuropathy, was treated only with intravenous immunoglobulin (IVIg), while the remaining patients received steroid therapy. Among the 18 patients, 38.9% (7/18) received steroids alone, 38.9% (7/18) underwent a combination of steroids and IVIg, 5.5% (1/18) received steroids along with oral immunosuppressive drug (ISD) mycophenolate mofetil (MMF), and 11.1% (2/18) were treated with a combination of steroids, IVIg, and oral MMF (**Figure 3B**). The administration methods and dosages of steroids varied among the patients. Most patients (70.6%, 12/17) were treated with high-dose MP intravenous pulses followed by oral tapering of steroids. A smaller group (11.8%, 2/17) received medium-dose MP (MDMP) intravenously (40 mg/day) for 1–2 weeks, followed by oral tapering (**Figure 3C**). A few patients were treated solely with intravenous or oral steroids. No patient received biologics such as rituximab. Most patients demonstrated significant recovery following treatment. However, Patient 16 succumbed to severe complications associated with teratoma, resulting in progressive deterioration.

## Discussion

In this case, we reported a young male diagnosed with anti-mGluR5 encephalitis, characterized by persistent low-grade fever for one month, followed by progressive symptoms of higher cortical dysfunction, including apathy, drowsiness, impaired memory, and disorientation. The patient also presented with frequent seizures, agitation, and aggressive behavior. Imaging studies, including MRI and CT of the brain, showed no significant abnormalities, while EEG revealed predominantly β-wave patterns with scattered bursts of short β rhythms. CSF analysis indicated significantly elevated white blood cell count and protein levels, and both serum and CSF tested strongly positive for mGluR5 antibodies. Comprehensive clinical history and relevant screening found no evidence of an associated tumor. Following intravenous methylprednisolone pulse therapy, the patient’s symptoms rapidly improved. To prevent relapse, he received regular rituximab infusions alongside a steroid taper regimen. Over the subsequent year, the patient remained in remission without any recurrence of symptoms.

mGluR5, a member of Group I mGluRs, couples with Gαq/11 proteins to activate phospholipase C, leading to the formation of diacylglycerol and inositol triphosphate, and subsequently activating protein kinase C. Additionally, mGluR5 can stimulate other downstream kinases such as extracellular signal-regulated kinase (ERK1/2) and protein kinase B (Akt) (4). Widely distributed across the CNS, mGluR5 plays a vital role in regulating excitatory neural networks, promoting neurogenesis, and facilitating synaptic plasticity associated with learning and memory. Recent studies suggest that mGluR5 is involved in the pathogenesis of various neurological disorders, including fragile X syndrome, Huntington’s disease, Parkinson’s disease, Alzheimer’s disease, and even stroke (14). Though commonly associated with limbic encephalitis, case reports and series increasingly reveal the clinical and radiological heterogeneity of anti-mGluR5 encephalitis. The condition encompasses a broader spectrum of manifestations, such as psychiatric disturbances, dystonia, and peripheral neuropathy. The correlation between clinical symptoms and the distribution or titer of mGluR5 antibodies remains unclear. A review of previous reports highlights cases where patients with negative CSF antibody exhibited symptoms of limbic encephalitis (4, 14), as well as instances of CSF antibody-positive patients showing features of peripheral demyelinating neuropathy (10).

Paraneoplastic encephalitis associated with tumors, particularly Hodgkin lymphoma, has traditionally been recognized as accounting for a significant subset of cases. This association is particularly pronounced in Western populations, as evidenced by the elevated expression of mGluR5 in Hodgkin lymphoma tissues (14), which may trigger autoimmune responses. However, most reported cases in China, including our own, involve patients without a history of tumors, suggesting potential regional or genetic differences in etiology. This underscores the need for further research to clarify the relationship between this condition and tumor presence. Nonetheless, the possibility of an association with malignancy cannot be entirely excluded. Among the 18 patients reviewed, 3 were diagnosed with tumors and 1 with HD. This underscores the importance of maintaining vigilance for tumor development in such cases, particularly neural-origin tumors and teratomas. Notably, among the 4 patients with concurrent tumors or HD, 3 exhibited dystonia—a symptom rarely observed in typical encephalitis. Therefore, clinical attention should be heightened, and further investigation into the connections between tumors, CNS autoantibodies, and movement disorders is warranted.

The clinical spectrum of anti-mGluR5 encephalitis is broad, and seizures are a common presenting feature, occurring in 38.9% (7/18) of our reviewed Chinese cohort. The semiology of these seizures is heterogeneous. In our case series, Patient 2 and 16 experienced focal seizures, while Patients 4, 9, and 11 had generalized tonic-clonic seizures (1, 6–13). Notably, Patient 7 presented with faciobrachial dystonic seizures (FBDS), a phenotype typically associated with anti-LGI1 encephalitis, highlighting the potential for overlapping symptoms (1). Furthermore, Patient 13 had absence seizures (8). Importantly, status epilepticus was identified in several severe cases (Our patient and Patients 4, 7, 9, 11), indicating that this is a potentially life-threatening complication of the disease. Although the characteristic extreme delta brush seen in anti-NMDAR encephalitis was not observed, early and aggressive immunotherapy remains the cornerstone for controlling the autoimmune-driven central nervous system excitability. This should be complemented by appropriate anti-seizure medications to manage acute symptoms and prevent further neuronal injury. EEG findings in autoimmune encephalitis are typically non-specific. While generalized slowing and epileptiform discharges are most frequently reported (6–13), our patient’s initial EEG, performed on the day of admission, showed a predominant beta-wave pattern. This finding is atypical for the disease process itself. We postulate that this pattern was not a direct manifestation of the encephalitis, as the patient had not yet progressed to status epilepticus at that time. Instead, it was most likely an effect of the continuous intravenous midazolam infusion administered for sedation and agitation control, as benzodiazepines are well-known to induce fast-frequency activity on EEG.

In addition to antibody testing, diagnostic tools such as MRI, EEG, and various CSF analyses play a crucial role in identifying anti-mGluR5 encephalitis. MRI findings in these patients are often nonspecific, resembling patterns seen in other central nervous system inflammatory disorders, but they typically correspond to areas of clinical relevance. While EEG and elevated CSF WBC counts demonstrate lower sensitivity compared to MRI, their diagnostic significance increases when abnormalities are observed alongside MRI results. In our case, the elevated CSF protein warrants closer attention, as CSF WBC levels normalized rapidly after treatment, whereas CSF protein levels declined more gradually, underscoring its more stable diagnostic relevance. Additionally, OCB analysis should be prioritized. Among patients undergoing OCB testing, including ours, the frequent presence of positive OCB patterns was observed. Specifically, Pattern 2 (and Pattern 3) strongly indicates intrathecal IgG synthesis, while Pattern 4, showing identical bands in CSF and serum, suggests a systemic inflammatory response with disruption of the blood-brain barrier. Combined with the IgG Index and the 24-hour intrathecal IgG synthesis rate, these findings offer valuable insights into the comprehensive assessment of CNS immune status.

Therapeutic outcomes in anti-mGluR5 encephalitis are generally favorable, with most cases achieving complete or partial recovery following immunotherapy and removal of underlying triggers, such as tumor resection or appropriate treatment. Though it is also important to note that one patient (No. 9) had a very short follow-up period of only 4 weeks, which limits the assessment of long-term outcomes and relapse risk in that individual case. During the acute phase, intravenous IVIg and steroids were used either alone or in combination to control disease progression. To prevent chronic relapse, oral steroid tapering and ISDs were administered. However, the management of our patient incorporated a prophylactic strategy with rituximab, a decision guided by an individualized assessment of his specific risk factors, including an exceptionally severe acute phase marked by refractory status epilepticus and aggressive behavior, coupled with notably high anti-mGluR5 antibody titers (1:640 in CSF), which are among the highest reported. Given the rarity of the condition and the possibility that small case series may underestimate relapse risk in such severe phenotypes, we opted for a proactive approach following first-line steroids. The single 800mg dose of rituximab was calculated based on the standard neuroimmunological dosing (375 mg/m²), corresponding to the patient’s body surface area (2.11 m²). This single infusion, intended for relapse prevention rather than acute symptom control, effectively achieved sustained CD20+ B-cell depletion. The patient maintained clinical remission throughout the steroid taper and after its discontinuation over one year of follow-up, justifying the decision not to administer further cycles. Nevertheless, given the relatively potent immunosuppressive effects and associated risks of adverse reactions with rituximab, its further application—particularly in elderly patients or those with concomitant tumors—requires thorough risk assessment. The treatment of anti-mGluR5 encephalitis, akin to other autoimmune conditions of the central nervous system, should emphasize tailored therapeutic strategies, particularly in cases with atypical clinical manifestations or comorbidities involving other CNS autoantibody-mediated diseases.

## Data Availability

The original contributions presented in the study are included in the article/**Supplementary Material**. Further inquiries can be directed to the corresponding author.
