# Supplementary material for: Clinical spectrum and outcomes of anti-metabotropic glutamate receptor 5 encephalitis in Chinese patients: a case report and literature review
Source: Front Immunol. 2025 Nov 6;16:1656832. doi: 10.3389/fimmu.2025.1656832 (PMC12631469; doi:10.3389/fimmu.2025.1656832)
Supplement: Supplementary file 1 [file Table1.docx]

Supplemental Table S1 Comprehensive autoimmune antibodies tested in the patient's serum and cerebrospinal fluid (CSF) using cell-based assay (CBA).

| Antibody Types (Antigen) | CSF CBA Result | Serum CBA Result |
| --- | --- | --- |
| N-methyl-D-aspartic acid receptor (NMDAR) | Negative | Negative |
| Α-amino-3-hydroxy-5-methyl-4-isoxazole-propionic acid receptor 1 (AMPAR1) | Negative | Negative |
| Α-amino-3-hydroxy-5-methyl-4-isoxazole-propionic acid receptor 1 (AMPAR2) | Negative | Negative |
| Leucine-rich glioma inactivated 1 (LGI1) | Negative | Negative |
| Contact protein related protein-2 (CASPR2) | Negative | Negative |
| Dipeptidyl‐peptidase‐like protein 6 (DPPX) | Negative | Negative |
| Anti-immunoglobulin-like cell adhesion molecule 5 (IgLON5) | Negative | Negative |
| Glutamic acid decarboxylase 65 (GAD65) | Negative | Negative |
| Metabotropic glutamate receptor 5 (mGluR5) | Positive (1: 640) | Positive (1: 160) |
| Glycine receptor α1 (GlyRα1) | Negative | Negative |
| γ-aminobutyric acid B receptor (GABA_B_R) | Negative | Negative |
| γ-aminobutyric acid A receptor α1 (GABA_A_Rα1) | Negative | Negative |
| γ-aminobutyric acid A receptor β3 (GABA_A_Rβ3) | Negative | Negative |
| γ-aminobutyric acid A receptor γ2 (GABA_A_Rγ2) | Negative | Negative |
| Dopamine D2 receptor (D2R) | Negative | Negative |
| Neurexin 3α | Negative | Negative |
| Glial fibrillary acidic protein (GFAP) | Negative | Negative |
| Myelin oligodendrocyte glycoprotein (MOG) | Negative | Negative |
| Aquaporin 4 (AQP4) | Negative | Negative |
| Hu | Negative | Negative |
| Yo | Negative | Negative |
| Ri | Negative | Negative |
| Titin | Negative | Negative |
| Recoverin | Negative | Negative |
| Protein kinase C γ (PKCγ) | Negative | Negative |
| Zic family member 4 (ZIC4) | Negative | Negative |
| Delta/Notch-like epidermal growth factor-related receptor (DNER) | Negative | Negative |
| Sry-like high mobility group box 1 (SOX1) | Negative | Negative |
| Ma1 | Negative | Negative |
| Ma2 | Negative | Negative |
| Amphiphysin | Negative | Negative |
| Crossveinless 2 (CV2) | Negative | Negative |
